# Combined Effect: Development and Physical/Biological Assessment of PVA/Chitosan Hydrogels Containing rhTGF-β1-Loaded PLGA Nanoparticles

**DOI:** 10.3390/gels12060510

**Published:** 2026-06-08

**Authors:** Aysun Çelik-Soysal, Sevinç Şahbaz, Ali Demir Sezer, Timuçin Uğurlu

**Affiliations:** 1 Department of Pharmaceutical Biotechnology, Faculty of Pharmacy, Bulent Ecevit University, 67600 Zonguldak, Turkey; 2 Department of Pharmaceutical Technology, Faculty of Pharmacy, Marmara University, 34668 Istanbul, Turkey; 3 Department of Pharmaceutical Biotechnology, Faculty of Pharmacy, Marmara University, 34668 Istanbul, Turkey

**Keywords:** hydrogel, PLGA nanoparticle, PVA, chitosan, rhTGF-β1, wound healing

## Abstract

Wound healing remains a persistent health problem with no definitive solution. It is crucial to characterize the complex wound healing process and the various growth factors, cytokines, and polypeptides involved. Transforming growth factor beta1 (rhTGF-β1) stimulates different cell types, providing multifunctionality in the wound healing process. Since proteins are sensitive to proteases, drug delivery systems are needed. Developed polymeric carrier systems are as important as the active substance. The carrier systems used in our study aim to contribute to wound healing in addition to the rhTGF-β1. We hypothesized that PLGA nanoparticles embedded in PVA/Chitosan (PVA/Chi) hydrogels could enhance the therapeutic effect of rhTGF-β1. PVA/Chitosan hydrogels were prepared by the freezing/thawing method. Several characterization studies (Fourier transform infrared spectroscopy (FT-IR), scanning electron microscopy (SEM), texture analysis, and cell culture) were performed to investigate the potential of the prepared formulations to enhance the therapeutic effect of rhTGF-β1. Hydrogel formulations reduced the inhibitory effect of rhTGF-β1 on keratinocytes. The H5 hydrogel exhibited a proliferative effect on fibroblast cells, which play a crucial role in wound healing, resulting in a 78.8% increase compared to the control. As the PVA content in the hydrogel formulations increased, bioadhesion and viscosity also increased. Although TGF-β1 inhibited keratinocytes, it induced migration of both NIH-3T3 and HACAT cell lines. The formulations developed exhibit the potential to improve the therapeutic efficacy of rhTGF-β1 in wound healing. A small amount of the protein can have the same therapeutic efficacy and fewer side effects because the developed polymeric carrier systems contribute to the therapeutic efficacy.

## 1. Introduction

Currently, wound healing remains an unsolved medical issue. Rapid wound healing is critical for patient welfare as well as treatment costs [1,2,3]. Wound healing is a systematic process involving many cellular mechanisms. The process begins with inflammation and hemostasis, followed by proliferation, maturation, and remodeling. Many growth factors, cytokines, and receptors are involved in this process [4]. Transforming growth factor beta 1 (TGF-β1) is a protein involved in this process. It is released by fibroblasts, keratinocytes, platelets, macrophages, T lymphocytes, and endothelial cells. TGF-β1 is a 25-kDa protein consisting of 112 amino acids [5,6,7]. TGF-β1 promotes collagen synthesis and the production of fibroblasts during the wound healing process. It reduces protease expression and contributes to the synthesis of protease inhibitors. It contributes to the chemotaxis and maturation of macrophages and keratinocytes. While reducing the proliferation of keratinocytes in vitro, studies have reported both increases and decreases in vivo [8,9,10]. Drug delivery systems are being developed to ensure the stability, distribution, and efficacy of TGF-β1 without degradation. Poly(lactic-co-glycolic acid) (PLGA) is formed by the polymerization of polylactic acid and polyglycolic acid monomers in different amounts [11,12,13,14]. PLGA offers several advantages, including biocompatibility, biodegradability, and the ability to sustain release. It also supports angiogenesis and the activation of procollagen. In our previous study, the in vitro effectiveness of PLGA nanoparticles (NPs) was investigated [4,15,16]. The use of PLGA NPs in topical applications has several limitations. Consequently, hydrogel formulations have been developed to incorporate PLGA NPs, and the impact of these carrier systems on overall efficacy has been investigated. Hydrogels are water-insoluble, water-swellable, and cross-linked three-dimensional networks of polymer chains produced by the reaction of one or more monomers. Cross-linking is essential for achieving mechanical strength and physical integrity by reducing water solubility [17,18,19,20]. Hydrogels offer numerous benefits in wound healing, including the ability to cover the wound surface by releasing moisture, ease of preparation, and biocompatibility with the selected polymer [12].

Polyvinyl alcohol (PVA) is a biodegradable, semi-crystalline synthetic polymer utilized in biotechnological fields, including tissue regeneration, wound dressing fabrication, and drug delivery systems [21,22]. PVA-based dressings exhibit advantageous properties such as biodegradability, biocompatibility, elasticity, high water absorption capacity, non-toxicity, and low cost [23]. The PVA backbone contains a secondary alcohol group attached to a linear carbon chain and displays differential chemical properties, as well as variable solubility and crystallinity, as a function of the degree of hydrolysis [24,25,26,27,28]. Chitosan (Chi) is a linear-structured natural polymer extensively utilized in hydrogel fabrication. The cationic character of chitosan enhances its dermal interaction and adhesion properties. Owing to the protonation of its amino groups, chitosan demonstrates solubility in dilute solutions of various organic and inorganic acids. Chitosan stimulates fibroblast proliferation, facilitates orderly collagen deposition, and accelerates cutaneous wound healing by inhibiting scar formation, attributed to its gradual degradation into N-acetyl-β-D-glucosamine. Beyond tissue repair, chitosan augments the activity of leukocytes, fibroblasts, and macrophages through enhanced granulation tissue formation [29,30]. Nevertheless, chitosan-based hydrogels exhibit suboptimal mechanical properties and swelling capacities. These drawbacks can be ameliorated by employing crosslinking strategies during hydrogel formulation. Consequently, crosslinking chitosan with PVA enhances both the mechanical integrity and swelling behavior of the resulting hydrogels [29,30,31,32,33,34].

Additionally, PVA/Chi hydrogels are preferred due to their effectiveness against Gram-negative and Gram-positive bacteria [35]. These hydrogels are prepared using the freeze-thawing method, which eliminates the need for cross-linking agents that may cause toxicity [35,36,37]. Via this method, polymer chains exhibit substantially stronger interactions, thereby resulting in a stable hydrogel architecture with tunable mechanical properties [28]. Here, we present an evaluation of the characterization of PVA/chitosan hydrogels. Our goal was to determine the effectiveness of the formulations in terms of cell toxicity, proliferation, and migration using two different cell lines that are crucial for cutaneous wound healing. Additionally, we examined the impact of the formulations created in our study on the wound healing process, both independently and in conjunction with the active ingredient. Moreover, it has been demonstrated that peptide-protein active components, which are both expensive and unstable, exhibit efficacy even at low concentrations when used in conjunction with functional polymers.

## 2. Results and Discussion

### 2.1. Characterization of Formulations

#### 2.1.1. Size, Zeta Potential, PDI, and Encapsulation Efficiency of F NPs

A double-emulsion solvent evaporation method was selected for the preparation of the nanoparticles (NPs) due to the hydrophilic nature of the TGF-β1 protein. The characterization features of the F NP, which was optimized in our previous work with PLGA nanoparticles, are as follows: zeta potential −27.17 ± 3.25 mV; particle size 274.76 ± 1.50 nm; polydispersity index 0.317 ± 0.01; and encapsulation efficiency of 99.52 ± 0.12% (the sensitivity of the ELISA test is 3.3 pg/mL) [4]. The use of PLGA as a polymer offers numerous advantages. PLGA is a copolymer that contains different ratios of lactic and glycolic acid, promoting angiogenesis through lactate induction and accelerating healing in injured areas. PLGA with a (L:G 50:50) 503H acid terminal was selected. The carboxylic acid chain of PLGA enhances the interaction with the protein, allowing the protein to enter the polymer nanoparticle more effectively, and as a result, is thought to provide high encapsulation efficiency [4,15,16].

The hydrogels were fabricated by performing two freeze–thaw cycles. This approach was selected to mitigate the adverse effects of excessive cross-linking—typically induced by a higher number of cycles—on the water retention and release behaviors of the matrices [38,39]. Despite limitations in terms of industrial-scale production volume and long cycle times, the freeze–thaw method is frequently preferred because it is inexpensive and eliminates toxic chemical crosslinkers [40,41].

#### 2.1.2. Scanning Electron Microscopy

**Figure 1:**
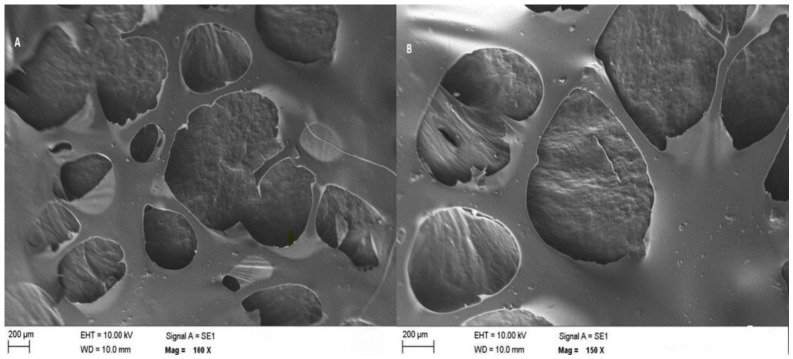
SEM images of PVA/Chi (H3) (**A**) and PVA/Chi/PLGA NP (H5) (**B**) hydrogel formulation.

SEM was used to characterize the morphological properties of the hydrogels. The surfaces of the PVA/chitosan hydrogels exhibit porosity resulting from the freezing process (Figure 1) [40,42,43]. As reported in our previous study, the morphological SEM analysis of the F nanoparticles was detailed elsewhere [4]. Scale bars and magnification information are presented directly below each SEM image.

#### 2.1.3. Fourier Transform Infrared Spectroscopy

**Figure 2:**
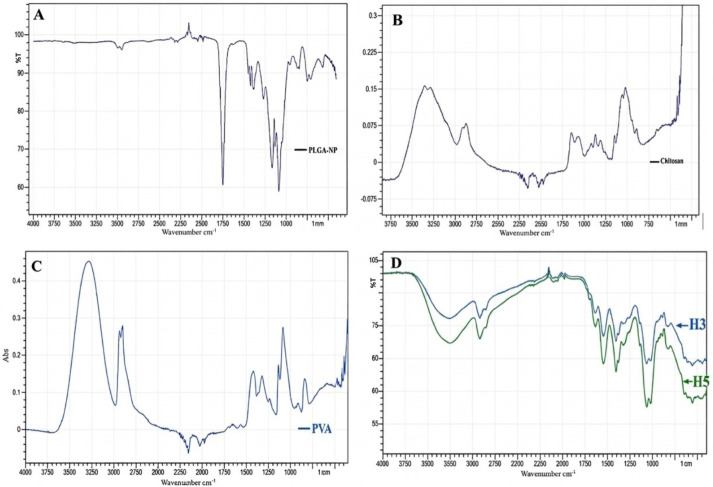
FT-IR. Fourier transform infrared spectra of F NP (**A**), Chitosan (**B**), PVA (**C**), PVA/Chi (H3) and PVA/Chi/PLGA NPs (H5) (**D**).

FT-IR analysis was performed to characterize the PVA/Chi hydrogels and PLGA NPs. Nanoparticle structures were characterized, demonstrating the interaction and compatibility between chitosan and PVA polymers in the synthesized hydrogels [4,44]. The FTIR spectra are shown in Figure 2. The interactions between the drug and the polymers are shown in Figure 2A. The peaks, including OH stretching (3560–3450 cm^−1^), -CH stretching (3000–2855 cm^−1^), carbonyl -C=O stretching (1780–1720 cm^−1^), and C-O stretching (1310–1160 cm^−1^), were visible in the PLGA 50:50 nanoparticles [4,45]. The FTIR spectrum of chitosan is shown in Figure 2B. The saccharin structure is represented by the peaks at around 893 cm^−1^. The bending vibration in the C-H group is characterized by the absorption peaks at 1475–1445 cm^−1^. In the amine group, the absorption peak at 1645 cm^−1^ has been attributed to the -C=O group. The C=O stretching vibration in CH is observed as a broad peak 1020–1070 cm^−1^. The signal located at 1151 cm^−1^ is indicative of the glycosidic linkage’s -C-O-C group. The PVA sample’s FTIR spectrum is displayed in Figure 2C. Here, significant hydroxyl bands characteristic of free alcohol (unbound OH stretching band at ν = 3450–3225 cm^−1^) are detected in conjunction with a broad C-H alkyl stretching band (v = 2855–3000 cm^−1^). It has been demonstrated that the C-O stretching in pure PVA occurs at 1125–1080 cm^−1^ [46,47]. The FTIR spectra of the hydrogel containing PLGA and chitosan-PVA were assessed, as shown in Figure 2D. The outcome demonstrated the high compatibility of chitosan and PVA polymers in the synthesized hydrogels. According to Javadzadeh et al., a peak that occurred around 3450–3225 cm^−1^ indicated the presence of hydrogen bonding between PVA polymers and chitosan, which caused OH/NH_2_ stretching [47]. Other peaks, such as those at 1707, 1638, and 1475–1415 cm^−1^, are related to the absorption of the NHCOCH_3_ group, the stretching of carbonyl groups, and the presence of C–H bonds in the PVA/chitosan polymer chains, respectively. The FTIR spectrum indicates that the PVA/Chi hydrogel structure, containing PLGA nanoparticles, has been preserved. The asymmetric CH_2_ stretching and C-H bond of the chitosan/PVA and chitosan chains, respectively, are the sources of the absorption peaks that were seen at 2975–2845 cm^−1^ and 2870–2840 cm^−1^ in the H3 and H5 hydrogels, respectively. The preservation of the PVA/Chi hydrogel structure in the H5 formulation containing the PLGA nanoparticle was confirmed by the FT-IR spectrum, which showed the presence of the characteristic peak of the PLGA nanoparticle structure bond at 1720–1695 cm^−1^ (C=O) [48,49,50,51].

#### 2.1.4. In Vitro Release Studies of Formulations

**Figure 3:**
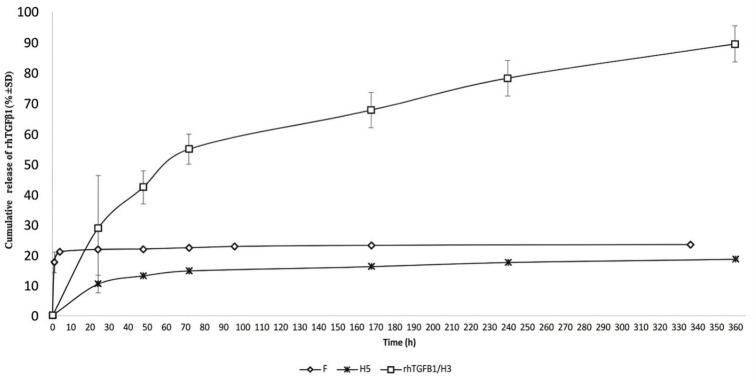
Comparison of the release of rhTGF-β1 protein from F np, H5 and rhTGF-β1/H3 hydrogel (PBS buffer, pH 7.4, 37 °C).

The H5 hydrogel demonstrated a characteristic biphasic release profile. Within the first 24 h, cumulative protein release from the nanoparticle-loaded hydrogel (H5) was 10.71 ± 3.02%, whereas the F NPs and the rhTGF-β1/H3 formulation released 22.22 ± 0.57% and 29.57 ± 17.84% of the protein, respectively. Both the burst effect and the long-term release kinetics of the F NPs and the H5 hydrogel exhibited significant variations compared to the rhTGF-β1/H3 formulation (*p* < 0.05). By day 15, the H5 hydrogel formulation achieved a sustained release, discharging 19.08 ± 0.29% of the encapsulated rhTGF-β1. According to the release study, the initial burst effect was determined as 18% for the F NPs, 29.57% for the rhTGF-β1/H3 formulation, and 10.71% for the H5 hydrogel. Notably, the incorporation of F NPs into the hydrogel matrix significantly mitigated the burst release of rhTGF-β1 (*p* < 0.05) and enhanced its sustained release profile (Figure 3) [25,52].

#### 2.1.5. Bioadhesion Studies of Formulations

**Figure 4:**
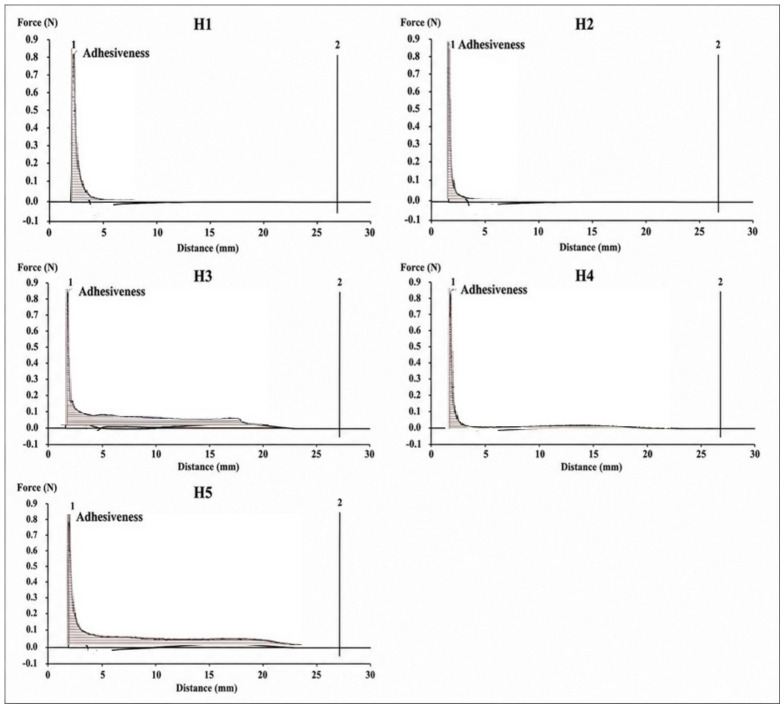
Work of bioadhesion graphs of H1, H2, H3, H4, and H5 formulations. The work of bioadhesion is the area under the curve between 1 and 2 (AUC_1-2_) (*n* = 3).

The F NPs exhibited a bioadhesion value of 0.12 ± 0.08 mJ/cm^2^, indicating a need for further enhancement despite being functional for topical delivery. This limited adhesion is attributed to the electrostatic repulsion or minimal interaction between the negatively charged skin proteins and the negatively charged PLGA nanoparticles. Consequently, hydrogel formulations were developed to promote the interaction between the nanoparticle system and the wound tissue, thereby supporting the healing process. The incorporation of chitosan provides the hydrogel matrix with cationic properties, which successfully facilitates ionic interactions within the wound microenvironment [4,53,54]. The bioadhesive properties of five distinct hydrogel formulations were evaluated using chicken skin as a tissue model. The in vitro bioadhesion work values for H1, H2, H3, H4, and H5 were determined to be 0.072 ± 0.007; 0.218 ± 0.011; 0.509 ± 0.032; 0.430 ± 0.045 and 0.547 ± 0.010, respectively (Figure 4). Among the blank hydrogels, the formulation exhibiting the highest bioadhesion capacity was H3, while its nanoparticle-loaded counterpart was designated as H5. The comparative bioadhesion profiles for each gel formulation are illustrated in Figure 4.

The bioadhesive activity of nanoparticles has been comprehensively addressed in our previous study [4]. The aim was to enhance the limited bioadhesion properties of the optimized F NPs for topical application by combining them with a hydrogel. The bioadhesive properties of hydrogels offer several advantages in wound treatment. The goal is to increase drug concentration in the target area by prolonging residence time at the application site, thus providing a longer duration of action and reducing dosing frequency. Chitosan, a natural polymer, exhibits bioadhesive properties on its own [55]. The incorporation of various polymers with chitosan further enhances this bioadhesiveness. The bioadhesiveness of hydrogels prepared with varying PVA concentrations increased significantly, exhibiting a linear relationship with PVA concentration. Although the H4 hydrogel, composed of chitosan alone, displayed higher bioadhesiveness than H1 and H2, a significant increase was observed in H3, which contained 10% PVA (*p* < 0.05). This finding is supported by studies on hydrogels containing high concentrations of PVA [56]. The PVA/chitosan hydrogel (H5) containing F NPs significantly increased bioadhesiveness to tissue, enhancing its properties by interacting with negatively charged tissue proteins [54].

#### 2.1.6. Mechanical Properties of PVA/Chitosan Hydrogels

Hydrogels are evaluated for their potential applications based on their mechanical properties, including hardness, adhesiveness, elasticity, cohesiveness, and resilience (Table 1). The mechanical properties of PVA/Chi hydrogels were assessed using a texture analyzer, which measures the force required to deform the hydrogels and thereby determines the hardness value. Low gel hardness indicates that the gels can be easily removed from tubes and applied to the desired area with minimal effort. In contrast, a high hardness value can reduce the amount of time the gel formulation remains on the wound. Therefore, an appropriate hardness value is necessary for effective wound treatment. The hardness of the hydrogels increased with increasing PVA content, with H3, containing 10% PVA, exhibiting the highest hardness value of 0.455 ± 0.077 N, a significant result compared to the other hydrogels (*p* < 0.05) [57]. Hydrogels prepared with chitosan alone have limited mechanical properties and flexibility. High PVA concentrations contribute to the improvement of these properties [58]. The hydrogel containing nanoparticles (H5) exhibited a significant decrease in hardness compared to H3 but an increase compared to H1, H2, and H4 (*p* < 0.05). This decrease is attributed to reduced cross-linking and a lower hardness value in the cross-linked regions of the gel [59,60,61].

**Figure 5:**
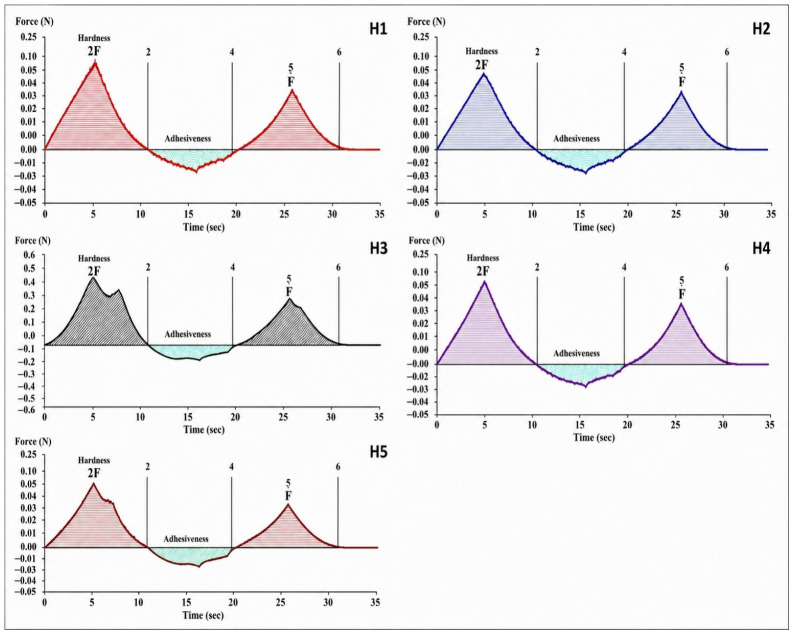
Texture profile analysis graphs of PVA/Chitosan hydrogels (H1, H2, H3, H4, H5) (*n* = 3).

The optimal duration of hydrogel adhesion is crucial for reducing treatment time and ensuring patient compliance. According to a previous study, the concentration and molecular weight of chitosan can significantly impact hydrogel adhesion [62] (Figure 5). This study focuses on the preparation of PVA/chitosan hydrogels using medium-molecular-weight chitosan (400 kDa). The adhesion of the hydrogels increased with higher PVA ratios, with the highest adhesion observed in the H3 hydrogel, which contained a 10% PVA ratio. However, increasing the PVA concentration from 2.5% to 5% did not significantly affect gel adhesion (*p* > 0.05). The H4 hydrogel prepared with chitosan alone exhibited higher adhesion than the H1 and H2 formulations (*p* < 0.05). The H5 hydrogel containing nanoparticles demonstrated a therapeutically effective adhesion value of 0.243 ± 0.013 N·mm [62].

In addition to adhesion, the elasticity of the hydrogels was also evaluated. Elasticity is defined as the gel’s ability to return to its original shape after deformation. The results showed that the elasticity of the hydrogels decreased with increasing PVA concentration, with the highest elasticity observed in the H1 hydrogel (0.957 ± 0.010) (*p* < 0.05). Although H3 exhibited the highest adhesion, it had the lowest numerical elasticity value (0.918 ± 0.017). There was an increase in elasticity in H3 compared to H5; however, this difference did not reach statistical significance (*p* > 0.05). Strong bioadhesion between the polymer and the mucosal surface necessitates high elasticity, since the mechanism of bioadhesion is dependent on the elasticity of the polymer chains [63]. The findings of this study support previous research indicating that PVA-containing hydrogels exhibit high adhesion properties due to the crystallinity of PVA [58].

While the presence of PVA in PVA/chitosan hydrogels has a positive effect on flexibility [57,64], our results also indicate compatibility in terms of bioadhesion. The elasticity of PVA hydrogels is influenced by the crystallinity of PVA, which increases with increasing PVA concentration, resulting in enhanced flexibility [60,65]. Cohesiveness is a crucial parameter for topical hydrogel applications and is evaluated as the attractive force between molecules within the gel, which affects adhesion [66]. The cohesive value decreased as the PVA concentration in the hydrogels increased. The highest cohesive value was observed in the hydrogel formulation prepared with chitosan alone (H4; 0.665 ± 0.014), while the cohesive value of the gel containing nanoparticles (H5; 0.503 ± 0.070) increased compared to H3 (0.453 ± 0.043); however, this difference did not reach statistical significance (*p* > 0.05). Sezer et al. reported that the cohesiveness of hydrogels increased with increasing chitosan concentration [62]. The absence of PVA and the amount of chitosan in the prepared hydrogel formulations explain why the H4 formulation exhibited the highest cohesive properties. Furthermore, the flexibility of the H4 hydrogel (without PVA) exhibited the highest value (0.256 ± 0.042). No significant increase or decrease was observed in the other hydrogel formulations containing PVA.

#### 2.1.7. Viscosity Measurements of PVA/Chitosan Hydrogels

The viscosity values of all hydrogel formulations are presented in Table 1. According to the findings, the H3 formulation exhibited the highest viscosity, measuring 21,300.0 ± 5208.4 cP. The H5 formulation, which contained nanoparticles, had the second-highest viscosity at 19,433.0 ± 365.0 cP.

In PVA/chitosan hydrogels, viscosity increased linearly with increasing PVA concentration due to the constant chitosan ratio. Only the H4 hydrogel had a higher viscosity value than the H1 and H2 hydrogel formulations, which contained 2.5% and 5% PVA, respectively (*p* < 0.05). The H3 hydrogel with 10% PVA was statistically more viscous than the H4 hydrogel (*p* < 0.05). A decrease in viscosity was observed in the H5 hydrogel compared to the H3 formulation; however, this decrease was not statistically significant (*p* > 0.05). Increased chitosan content resulted in higher gel density and increased viscosity. Similarly, high PVA content is known to increase viscosity through hydrogen bonding interactions within the polymer. The increase in viscosity of the PVA/chitosan hydrogel formulations is attributed to the rise in intra-chain hydrophobic bonds in chitosan and intermolecular interactions between PVA and chitosan molecules [67].

#### 2.1.8. Water Absorption Capacity of PVA/Chitosan Hydrogels

**Figure 6:**
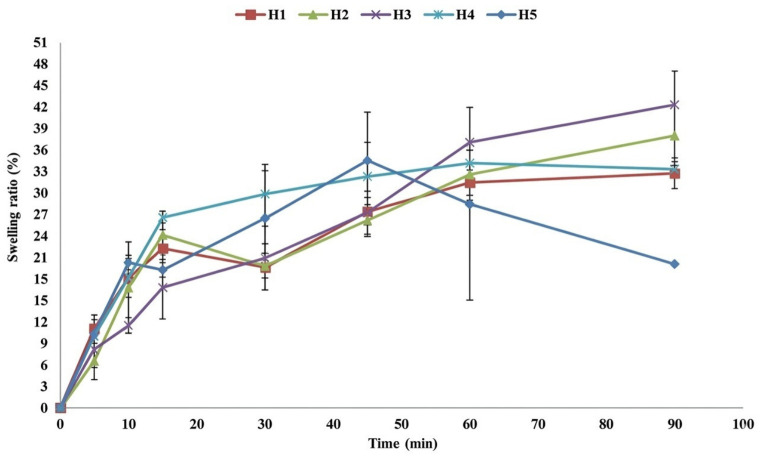
The effects of concentration of PVA on the water absorption capacity of hydrogel formulations (*n* = 3), (PBS buffer, pH 7.4, 37 °C).

The water uptake capacity of PVA/chitosan hydrogels depended on the concentration of PVA polymer, which increased the water retention capacity and swelling rate of the hydrogels (*p* < 0.05) (Figure 6). Increasing the PVA concentration from 2.5% to 5% resulted in a 5% increase in water retention capacity, and increasing it from 2.5% to 10% resulted in a 10% increase. However, there was no significant difference in swelling ratios between the formulations with 2.5% and 5% PVA and the H4 hydrogel prepared with chitosan alone (*p* > 0.05). The 10% PVA concentration exhibited a significant increase in water retention compared to all other hydrogels (*p* < 0.05) (Table 1) [39].

The hydrophilicity of the polymer network also increased with increasing PVA concentration, which in turn increased the swelling rate [51]. The H5 formulation exhibited a faster swelling rate and a wider cross-linking range, which can be attributed to the nanoparticles embedded within the hydrogel. However, this was limited by additional cross-links between the PLGA nanoparticles and the electron-rich oxygen and nitrogen atoms in the polymer chains, which reduced the hydrogel’s ability to penetrate water and its overall swelling behavior [26,59]. The water retention capacity of the nanoparticle-containing hydrogel decreased from 42% to 20% compared to the nanoparticle-free hydrogel.

#### 2.1.9. In Vitro Cytotoxicity Studies

**Figure 7:**
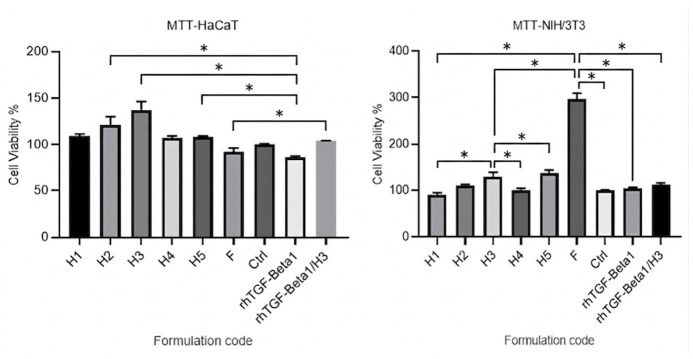
MTT cell viability results of HaCaT and NIH 3T3 cell lines (*n* = 3). The control (non-treated) group includes HaCaT and NIH 3T3 cells, respectively. Values are the mean of triplicate determination (*n* = 3) ± standard deviation and statistically significant at * *p* < 0.05.

For the MTT cytotoxicity assays, NIH-3T3 fibroblast and HaCaT keratinocyte cell lines were utilized, as they are widely established models for in vitro wound healing investigations. rhTGF-β1 protein has a proliferative effect on fibroblast cells and an inhibitory effect on keratinocytes [8,68]. The cytotoxic effect was evaluated using formulations containing 2.5 ng/25 µL of rhTGF-β1 protein per well, and both protein-containing and protein-free formulations were compared. The results indicated that none of the developed pharmaceutical formulations were cytotoxic; in fact, they promoted cell viability. In the cytotoxicity study on the fibroblast cell line, none of the formulations were found to be cytotoxic. For the PVA/chitosan hydrogel formulations, cell viability increased with increasing PVA ratio. Among the hydrogels, the H3 formulation exhibited a significant increase in cell viability compared to the control (29.4%) and the other formulations (*p* < 0.05). The current results, summarized in Table 2 and Figure 7, also demonstrate that the H2, H3, and H5 hydrogel formulations significantly increased cell viability compared to the control (*p* < 0.05). Although the H5 hydrogel showed an increase in viability compared to H3, the difference was not statistically significant (*p* > 0.05). The impact of PLGA nanoparticles on cell viability has been thoroughly investigated in a previous study [4]. The PVA/chitosan hydrogels were developed for the topical application of nanoparticles.

In the fibroblast cell line, the H5 hydrogel formulation significantly increased cell viability compared to the control (38.2%) and the natural rhTGF-β1 protein (33.5%) (*p* < 0.05), while also providing stability to the protein as a delivery system [69]. These findings are consistent with literature data [70]. A previous study demonstrated that F NPs are non-cytotoxic and enhance cell viability by 196.81% (*p* < 0.05) in the fibroblast cell line, an effect attributed to lactic acid induction by PLGA [4]. According to Chereddy et al., rhTGF-β1 and lactic acid act synergistically to achieve high cell viability in protein-loaded nanoparticles [71], a parallelism confirmed by our study. Consequently, F nanoparticles were incorporated into the hydrogel formulation.

The developed formulations, in addition to the rhTGF-β1 protein, were believed to enhance cell viability, protect the protein from degradation by proteases and nucleases in the cellular environment, and contribute to its stability [48]. In the toxicity study on keratinocytes, the naked rhTGF-β1 protein exhibited the lowest cell viability (86.53 ± 1.19) due to its inhibitory effect on these cells [68]. A statistically significant increase in cell viability was observed with increasing PVA content in the hydrogels (*p* < 0.05). The H4 hydrogel formulation exhibited lower cell viability than those containing PVA [58]. The H2 and H3 hydrogel formulations significantly increased cell viability compared to the control (*p* < 0.05). This can be attributed to the hydrophilicity and high water absorption capacity of the PVA polymer at high concentrations [72]. The H4 and H5 hydrogels did not show a significant increase compared to the control (*p* > 0.05). F NPs were shown to significantly increase viability by reducing the inhibition of keratinocytes involved in the wound healing process (*p* < 0.05) [4,7], which is consistent with the BrdU cell proliferation study.

#### 2.1.10. BrdU Cell Proliferation Assay

According to the BrdU assay results, fibroblast proliferation was significantly enhanced in the hydrogel formulation groups relative to the control and free rhTGF-β1-treated groups (*p* < 0.05) (Table 3). This proliferative effect can be attributed to the ability of chitosan to stimulate cell proliferation by decreasing protein degradation through its binding to serum factors in vitro, as well as its ability to enhance the activity of rhTGF-β1 by promoting cell adhesion of PVA/chitosan hydrogels [73,74]. The rhTGF-β1 protein alone showed a non-significant increase of 6.7% compared to the control (*p* > 0.05). Although cell proliferation increased with increasing PVA ratio in the hydrogel formulations, no significant linear correlation was observed (*p* > 0.05). The H3 formulation exhibited a statistically significant superiority over the H1, H4, and H5 groups (*p* < 0.05), a phenomenon directly attributed to it possessing the highest PVA concentration among the formulations [58]. Furthermore, the nanoparticle-loaded H5 hydrogel demonstrated a remarkable capacity for tissue regeneration, inducing a substantial increase in NIH-3T3 cell proliferation compared to the control and free rhTGF-β1 protein groups by 78.8% and 72.1%, respectively (*p* < 0.05). The F NPs exhibited a statistically significant enhancement in cell proliferation compared to the control and free rhTGF-β1 protein groups, yielding increases of 92.6% and 85.9%, respectively (*p* < 0.05). This proliferative effect is primarily attributed to a synergistic mechanism involving both the intrinsic mitogenic activity of rhTGF-β1 on fibroblasts and the lactate induction mediated by the degradation of PLGA nanoparticles [4,71,75]. Compared to the control group, the H5 hydrogel showed a proliferative effect of 78.8%, while F NPs showed a proliferative effect of 92.6%, supporting the prolonged release effect of the H5 hydrogel observed in the in vitro release study. Among the protein-containing formulations, rhTGF-β1/H3, F NPs, and the H5 hydrogel exhibited the highest proliferative effects in the NIH-3T3 cell line, respectively. The release study further supports these findings. The hydrogel formulations demonstrated their suitability for topical application based on the various parameters discussed above [26,31].

**Figure 8:**
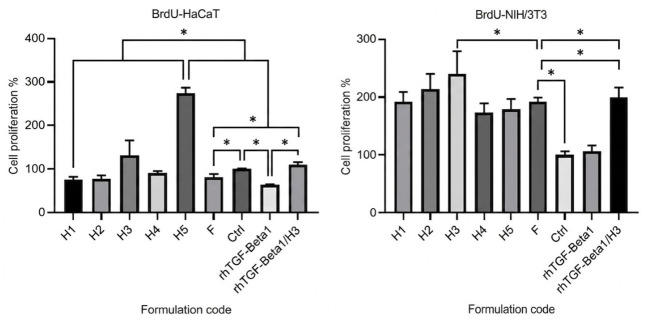
BrdU cell proliferation results of HaCaT and NIH 3T3 cell lines (*n* = 3). The control (non-treated) group includes HaCaT and NIH 3T3 cells consecutively. The cell proliferation ratio of the control group was accepted as 100%. Values are the mean of triplicate determination (*n* = 3) ± standard deviation and statistically significant at * *p* < 0.05.

As illustrated in Figure 8, the F NPs are postulated to promote keratinocyte proliferation by actively counteracting the inhibitory effects of free rhTGF-β1 [75]. The proliferative effect of the H5 hydrogel was found to be significantly greater than that of F NPs (*p* < 0.05), with an increase of 191.4 ± 13.57% compared to F NPs. This can be attributed to the combined proliferative effect of the PVA/chitosan hydrogel system [26,76]. Our MTT assay results on the HaCaT cell line support the findings of the cell proliferation study. In that study, the H3 hydrogel demonstrated a statistically significant increase in proliferation compared to the other hydrogel formulations (*p* < 0.05), with a proliferative effect of 31.3%. This is attributable to the fact that it does not contain rhTGF-β1 protein and therefore lacks its inhibitory effect [8]. Studies by Faler and colleagues have demonstrated that rhTGF-β1 exerts an inhibitory effect on keratinocytes while promoting migration in these cells [68]. The H5 hydrogel exhibited a significant (*p* < 0.05) and high proliferative effect (173.4 ± 13.57) compared to all other PVA/chitosan hydrogels and the control group, indicating that the previously described carrier systems contributed to the increased efficacy [71].

#### 2.1.11. In Vitro Wound-Healing Assay (Scratch Assay) and Wound Closure

**Figure 9:**
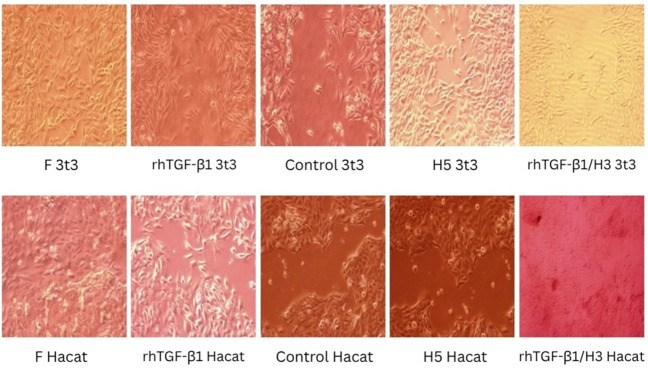
Scratch test (HaCaT and NIH-3T3 cells). Evaluation of the effectiveness of the selected formulations on wound closure after 48 h (*n* = 3).

Our findings demonstrated that HaCaT and NIH-3T3 cells were more effective in wells containing the developed formulations compared to the control group and the group containing native rhTGF-β1. The ability of PLGA nanoparticles (F NPs) and PVA/chitosan hydrogels to accelerate wound healing was evaluated using a scratch test. The selected formulations were F NPs, rhTGF-β1/H3, and H5 hydrogels. The results demonstrated that the developed formulations enhanced the proliferation and migration of HaCaT and NIH-3T3 cells to the wound site compared to the control medium and free rhTGF-β1 (Figure 9).

**Figure 10:**
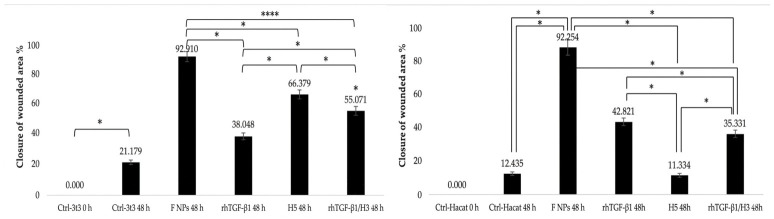
Wound closure assay %. Ctrl group (t: 0 h); Ctrl group (t: 48 h); F NPs group (t: 48 h); rhTGF-β1 group (t: 48 h); H5 group (t: 48 h); rhTGF-β1/H3 group (t: 48 h). Values are mean of triplicate determination (*n* = 3) ± standard deviation, and statistically significant at * *p* < 0.05 and **** *p* < 0.0001. In these experiments the same procedure was performed in HaCaT and NIH 3T3 cell lines.

Wound closure assays were conducted using HaCaT and NIH 3T3 cell lines to evaluate the effects of rhTGF-β1-containing formulations on wound closure and cell migration. The resulting wound closure percentages, along with their corresponding values, are presented in Figure 10.

In the NIH-3T3 fibroblast cell line, the F NPs demonstrated robust cell-migration capacity in in vitro wound-closure assays [77], establishing this formulation as the most promising candidate for effective wound healing. This performance is ascribed to the synergistic action of rhTGF-β1 combined with the PLGA nanoparticle-mediated lactate induction pathways [4,8,68,71]. The H3 formulation enhanced cell proliferation primarily due to its chitosan content, while the rhTGF-β1/H3 group exhibited a superior proliferative effect relative to the control group, driven by the synergistic structural benefits of both the protein and chitosan [78]. Conversely, the reduced migratory effect observed in the H5 group compared to the free F NPs can be attributed to the sustained and biphasic release profile of rhTGF-β1, which involves sequential diffusion from the nanoparticles into the hydrogel matrix, and subsequently from the hydrogel into the surrounding medium [52].

In the HaCaT cell line, the F NPs actively promoted cell migration; conversely, both the rhTGF-β1/H3 and H5 hydrogels exhibited limited migratory capacity and delayed wound closure relative to the control group. This restricted migration in the H5 hydrogel is primarily attributed to the delayed and sustained release of the encapsulated protein [52,77]. Furthermore, across both evaluated cell lines, free rhTGF-β1 protein alone exhibited lower migratory activity than the F NPs. This difference underscores the pivotal role of the developed nanoparticle delivery system in enhancing protein stability and reinforcing the proliferative effect [48,69], thereby ultimately facilitating rapid and effective wound healing.

The optimized FNP and H5 hydrogel formulations are considered promising candidates for wound treatment and are expected to serve as a foundation for more comprehensive studies. Although the originality of these formulations has been substantiated through multifaceted in vitro investigations, the absence of ex vivo and in vivo wound models on human skin remains a critical limitation. This shortcoming prevents the accurate recapitulation of immunological responses, cellular interactions, and tissue architecture, thereby severely restricting the translational potential of the developed therapies [79,80]. Therefore, as the next step, further investigation of the candidate formulations in vivo studies is envisaged.

## 3. Conclusions

In this study, we demonstrated the combined effect of the active ingredient, drug delivery system, and polymer selection on the therapeutic process. Our findings showed that PVA/chitosan hydrogels and F-PLGA nanoparticles significantly enhance the stability of rhTGF-β1, a protein effective in wound treatment. These delivery systems provide rapid, comfortable, and contamination-free healing while enabling long-term release suitable for chronic wound management. Furthermore, the study highlighted the therapeutic potential of polymers through lactate induction. In vitro evaluations confirmed the efficacy of the formulations, revealing proliferative effects and high cell viability. Consequently, the optimized FNP and H5 hydrogel formulations are promising candidates for wound treatment and will serve as a foundation for more extensive studies. Nevertheless, to generate more comprehensive and conclusive data, these promising therapeutic agents must be further supported by in vivo and/or ex vivo studies. Such an approach is essential for fully elucidating their mechanism of action and for facilitating their successful transformation into viable clinical products.

## 4. Materials and Methods

### 4.1. Materials

The following products were purchased: TGF-β1 ELISA kit (Enzo Life Sciences, Farmingdale, NY, USA); recombinant human TGF-β1 (rhTGF-β1) (Peprotech, Waltham, MA, USA); poly(vinyl alcohol) (PVA, 88% hydrolyzed, Mw 25,000) (Polysciences Inc., Warrington, PA, USA); and fully hydrolyzed PVA (Sigma P1763, St. Louis, MO, USA). These companies supplied the following materials: chitosan (medium), 190,000–310,000 Da, 75–85% deacetylated (Sigma, St. Louis, MO, USA); poly(L, D-lactic-co-glycolic acid) (PLGA, RG503H), Boehringer Ingelheim, Ingelheim am Rhein, Germany; and dichloromethane (DCM), Merck, Darmstadt, Germany. Our study employed the following instruments: 3-(4,5-dimethylthiazol-2-yl)-2,5-diphenyltetrazolium bromide (MTT) (Roche, Mannheim, Germany), BrdU (5-bromo-2′-deoxyuridine) cell proliferation test (Merck Millipore, Burlington MA, USA), NIH 3T3 (ATCC-CRL-1658), and HaCaT (ATCC-PCS 200-011) cell lines.

### 4.2. Preparation of PLGA Nanoparticles

**Figure 11:**
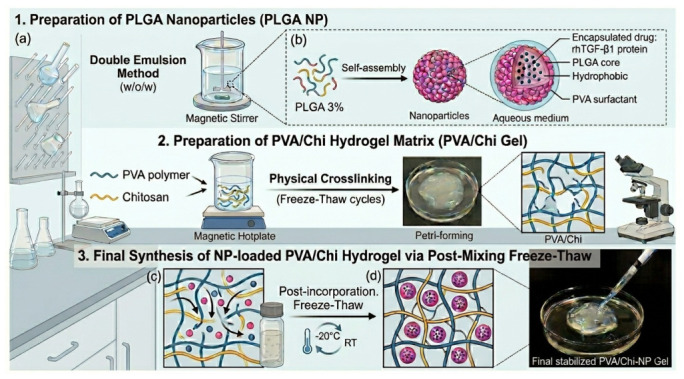
Synthesis of F NPs and PVA/Chi/PLGA NPs hydrogels. (**1**) Preparation of PLGA Nanoparticles (PLGA NP): (**a**) Formulation via the w/o/w double emulsion method using a magnetic stirrer, and (**b**) self-assembly of 3% PLGA into nanoparticles encapsulating rhTGF-β1protein within a hydrophobic PLGA core and stabilized by a PVA surfactant. (**2**) Preparation of PVA/Chi Hydrogel Matrix: Physical crosslinking of PVA polymer and chitosan blends via cyclic freeze-thaw methodology. (**3**) Final Synthesis of NP-loaded PVA/Chi Hydrogel via Post-Mixing Freeze-Thaw: (**c**) Post-incorporation of the prepared PLGA NPs into the hydrogel network, and (**d**) final stabilization of the composite system (Final stabilized PVA/Chi-NP Gel) through subsequent freeze-thaw cycles.

As in our previous study, PLGA nanoparticles were prepared using the double-emulsion solvent evaporation method [4]. To prepare a 3% PLGA solution, a quantity of PLGA was dissolved in DCM. 1 µg/10 µL of rhTGF-β1 was added and homogenized. Subsequently, the mixture was added to 50 mL of a 1% PVA solution and homogenized (w/o/w). The mixture was stirred using a magnetic stirrer to remove any residual DCM. The nanoparticles were separated by centrifugation at 12,000 rpm for 20 min. Thereafter, the nanoparticles were washed three times with deionized water and lyophilized using a Lyovac GT 2E (Steris, Tuttlingen, Germany) (Figure 11).

### 4.3. Preparation of PVA/Chitosan Hydrogels

The freezing and thawing procedure was used to create hydrogels of polyvinyl alcohol (PVA) and chitosan (Chi). PVA was dissolved in distilled water, while Chi was dissolved in water containing 1% acetic acid. A total of 50 mL of polymer mixture was prepared at a volumetric ratio of 4:1 and with a constant 2.5% chitosan concentration. The samples were stored at −20 °C for 12 h and then at room temperature (25 °C) for 6 h to complete one cycle of freezing and thawing. This procedure was carried out twice [58,81]. As a result of detailed characterization and cell culture studies in our previous study with PLGA nanoparticles, F-nanoparticles were identified and incorporated into PVA/Chi hydrogels in this study. Before the freeze–thaw cycle, 1 µg of rhTGF-β1 protein was added straight to H3 hydrogel to create rhTGF-β1/H3, and 100 mg of nanoparticles were added to H3 hydrogel to create H5 hydrogel. After preparation, the hydrogels were stored at +4 °C [25,56,82]. The hydrogel formulations are listed in Table 1.

### 4.4. Characterization of F (PLGA) Nanoparticles

In our earlier study with PLGA nanoparticles, detailed characterization analyses were performed, and the results were discussed. The primary analyses of PLGA nanoparticles include size measurement, zeta potential, polydispersity index, encapsulation efficiency, SEM morphological evaluation, FT-IR spectroscopy, DSC analysis, and release study. As a result of these analyses, F-coded nanoparticles were included in the PVA/Chi hydrogel study [4].

### 4.5. Characterization of Hydrogels

#### 4.5.1. SEM Analysis

Scanning electron microscopy (SEM) (Carl ZEISS, EVO 40, Oberkochen, Germany) was used to investigate the morphology of the formulations. The samples were coated with a gold-palladium layer after being placed on a metallic surface. The observations were made at a voltage of 10 kV [4,33].

#### 4.5.2. FTIR Analysis

Fourier transform infrared spectroscopy (FT-IR) (Thermo) was performed at 4 cm^−1^ resolution to verify the evolution of all formulations. All formulations were lyophilized using a Lyovac GT 2E (Steris, Tuttlingen, Germany). In the range of 4000–650 cm^−1^, the FTIR spectrum was obtained [4,33].

#### 4.5.3. Release Study

1 mL of PBS (pH 7.4) was added to the F NP, H5, and rhTGF-β1/H3 formulations containing 10 ng rhTGF-β1, and samples were collected on days 1, 2, 3, 7, 10, and 15 in a 37 °C water bath [83]. After centrifuging the samples for 10 min at 14,000 rpm, the supernatants were collected and stored at −20 °C. The incubation was then continued by adding fresh PBS until the next sampling time. The amount of rhTGF-β1 released from the hydrogels was determined using an ELISA kit [56,82]. The optimized F-coded NP was selected from the PLGA nanoparticles developed in our previous study [4]. The new formulation was formed by embedding it into the optimized hydrogel in this study, and an in vitro release comparison was performed.

#### 4.5.4. Bioadhesion Study

Bioadhesion studies of nanoparticles were carried out in detail in our previous study [4]. The TA.XT Plus Texture Analyzer (Stable Micro Systems, Godalming, Surrey, UK) was used for bioadhesion studies of PVA/Chitosan hydrogels, employing a p0.5 Perspex probe (12.5 mm diameter) with a 5 kg load cell. The tissue model used was all the fat and debris removed from the excised chicken back skin [62,84]. The excised chicken skin was attached to the bottom surface of the p0.5 Perspex probe, and equal amounts of the hydrogels were weighed and placed in 6-well plates. The probe holding the skin was lowered onto the surface of the hydrogels at a constant speed of 0.1 mm/s and with a contact force of 0.5 N, applied with a trigger force of 0.01 N. The probe was moved vertically upwards at a speed of 0.2 mm/s after staying in contact for 120 s. The Texture Exponent 6.1.27.0 software package of the instrument was utilized to compute the area under the curve (AUC) based on the force-distance plot as a measure of adhesion work. Every experiment was run three times. The work of bioadhesion per area was calculated using the formula below [53,54].

$$work\;of\;bioadhesion\;\left(mJ.{cm}^{-2}\right)=\frac{AUC}{\pi r^{2}}$$

$\pi r^{2}\;=$ the mucosal surface being in contact with the hydrogel.

#### 4.5.5. Mechanical Study of PVA/Chitosan Hydrogels

The mechanical properties of hydrogels were evaluated using a TA-XT Plus Texture Analyzer (Stable Micro Systems, Godalming, Surrey, UK), equipped with a p10 Perspex probe (10 mm diameter) and a 5 kg load cell. The test parameters were set to a test speed of 2.00 mm/s, a distance of 15.00 mm, a 2 s delay period between two compressions, and a trigger force of 0.010 N. The hydrogel formulations were placed in a universal bottle (50 mL) and subjected to ultrasonic treatment in a water bath for 30 min at a temperature of 25 °C to remove air bubbles. Each sample was measured in triplicate. The Texture Exponent 6.1.27.0 software package was used to collect and calculate the data. The hardness, adhesiveness, and cohesiveness of the hydrogels were determined based on the force-time graph. Hardness was defined as the maximum force or the maximum peak force relative to the initial deformation. Adhesiveness, indicated by the negative force area during the first compression cycle, was the work required to overcome the attraction forces between the hydrogel surface and the probe. Cohesiveness was calculated as the area under the force-time curve of the second compression divided by the area at the time of the first compression and the recovery time [62]. Elasticity is defined as the ratio of the time required to achieve maximum structural deformation on the second compression cycle to that on the first compression cycle when repeated compressions are separated by a predefined recovery interval [63]. Resilience is the sample’s capacity to recover from deformation, considering both speed and the forces applied. According to one definition, it is the ratio of the areas produced by the first compression cycle to the area between the *x*-axis crossing and the first probe reversal point [85].

#### 4.5.6. Viscosity Study of PVA/Chitosan Hydrogels

Hydrogels were prepared in 50 mL universal bottles. It was kept in an ultrasonic water bath to eliminate air bubbles. Viscosity studies were performed at 25 °C using a Brookfield DV-E viscometer (probe 6, 30 rpm). Each sample was measured three times [67].

#### 4.5.7. Swelling Study of PVA/Chitosan Hydrogels

The behaviour of hydrogels in terms of swelling is influenced by gel ionization and ionic strength. To investigate this, gravimetric swelling studies were conducted using a PBS buffer at pH 7.4. One gram of hydrogel was weighed and added to one ml of PBS gel at specific time intervals. After a certain period, any excess water was removed using blotter paper, and the weight was re-recorded. The water absorption capacity of the hydrogel was calculated as the difference in weight before and after absorption. This process was repeated three times for each sample [18,62].

SD (%) = ((W_t_ – W_0_)/W_0_) × 100

Wt = Weight of the hydrogel formulation at time t.

W_0_ = Weight of initial hydrogel formulation.

#### 4.5.8. MTT Assay

Fibroblast NIH 3T3 (ATCC-CRL-1658) and keratinocyte HaCaT (ATCC-PCS 200-011) cell lines originate from the American Type Culture Collection (ATCC) and were obtained from the Pharmaceutical Biotechnology Research Laboratory, Marmara University. The cytotoxicity of the hydrogels was assessed using the MTT assay on NIH3T3 and HaCaT cells. On 96-well plates (TPP, Trasadingen, Switzerland), 100 μL of complete media (DMEM with 10% FBS) was used to seed the cells at a density of 1 × 10^4^ cells per well. The cells were then incubated overnight. All hydrogels were suspended in DMEM medium. Additionally, 2.5 ng of protein was used to create formulations of F NP, H5, and rhTGF-β1/H3. The cells were cultured for twenty-four hours following the treatment. After that, each well received 10 microliters of MTT solution (5 mg/mL), and the cells were cultured for 12 h at 37 °C with 5% CO_2_. For a whole day, the formazan crystals of the living cells were left to disintegrate. Next, an ELISA reader for microplates was used to measure the absorbance at 550 and 690 nanometers. The percentage of cell viability in the control groups was reported [86,87]. These experiments were repeated three times.

#### 4.5.9. BrdU Assay

A colourimetric immunoassay, which measures BrdU incorporated during DNA synthesis, was used to determine the proliferation of NIH3T3 and HaCaT cells. The manufacturer’s instructions were followed for the BrdU assay. The seeding of the cells, addition of the formulation, and incubation time were the same as those for the MTT assay. A dual-wavelength 450/550 nm ELISA reader (Epoch Biotech, 13121287, Santa Clara, CA, USA) was used to calculate the absorbance of the plate. The experiments were repeated three times.

#### 4.5.10. In Vitro Wound Healing Assay (Scratch Assay)

The in vitro scratch assay is a well-established method used to quantitatively evaluate cell migration. In this method, a mechanical scratch is created in a confluent cell monolayer, and the rate of gap closure is monitored microscopically over time. The percentage of wound closure is calculated by comparing the remaining open area at the end of the incubation period (48 h) to the initial wound area (0 h). This quantitative parameter objectively demonstrates the efficacy of tested formulations or growth factors (such as rhTGF-β1) in promoting cellular migration and tissue regeneration [30,88].

Wound closure % = (A_0_ – A_t_) × 100/A_0_

A_0_: Initial area of the wound measured immediately after scratching (t = 0).

A_t_: Area of the wound at a specific later time point (t).

In six-well plates, HaCaT cells and NIH3T3 fibroblast cells were seeded at a density of 5 × 10^5^ cells/well in DMEM medium. The cells were allowed to grow until they reached a density of 70–80%. The next step was to use a sterile 200 µL pipette tip to make a scratch in each well. After removing the cells, the medium was replaced with PBS (pH 7.4), and the solution was rinsed twice. Selected formulations and positive control rhTGF-β1 were added to the medium. The in vitro wound healing model utilized optimized formulations of F NP, H5, and rhTGF-β1/H3 hydrogels, each containing 2.5 ng of protein per well. Images were taken using an inverted microscope equipped with an Olympus IX2 SLP camera system (Olympus CKX41, Tokyo, Japan) at the time of the first scratch and after 48 h. The cells were maintained at 37 °C and 5% CO_2_ throughout the recovery procedure, and the experiments were repeated three times [71,77].

#### 4.5.11. Statistical Analysis

Every outcome was displayed as means +/− standard deviation. A one-way analysis of variance (ANOVA) and the Newman–Keuls test for multiple comparisons were used in the statistical analysis. Differences between groups were deemed significant when *p* < 0.05. The statistical analyses were performed using GraphPad Prism (version 8.1).

## Figures and Tables

**Table 1:** Formulations of hydrogels’ mechanical properties, water absorption capacity, bioadhesion, and viscosity studies (*n* = 3).

Formulation Code	PVA%	Chi %	Containing PLGA np	Work of Bioadhesion (mJ/cm^2^ ± SD)	Viscosity (cps ± SD)	Hardness (N ± SD)	Adhesiveness (N.sec ± SD)	Elasticity ± SD	Cohesiveness ± SD	Resilience ± SD	Water Absortion Capacity (g ± SD)
H1	2.5	2.5	−	0.072 ± 0.007	6766.7 ± 450.8	0.052 ± 0.002	0.037 ± 0.007	0.957 ± 0.010	0.626 ± 0.012	0.178 ± 0.009	0.327 ± 0.022
H2	5	2.5	−	0.218 ± 0.011	8196.7 ± 57.7	0.089 ± 0.003	0.053 ± 0.009	0.934 ± 0.005	0.470 ± 0.032	0.202 ± 0.017	0.380 ± 0.042
H3	10	2.5	−	0.509 ± 0.032	21,300.0 ± 5208.4	0.455 ± 0.077	0.315 ± 0.026	0.918 ± 0.017	0.453 ± 0.043	0.137 ± 0.003	0.424 ± 0.047
H4	-	2.5	−	0.430 ± 0.045	15,233.3 ± 1615.0	0.098 ± 0.019	0.086 ± 0.020	0.940 ± 0.006	0.665 ± 0.014	0.256 ± 0.042	0.333 ± 0.010
H5	10	2.5	+	0.547 ± 0.010	19,433.0 ± 0.365	0.207 ± 0.037	0.243 ± 0.013	0.907 ± 0.006	0.503 ± 0.070	0.142 ± 0.008	0.200 ± 0.003

Note: −/+.

**Table 2:** % Cytotoxicity results of formulations (*n* = 3).

	% Cell Viability Average ± SD	
Formulations Code	NIH-3T3 Cell Line	HACAT Cell Line
F np	296.8 ± 12.79	92.6 ± 3.92
rhTGF-β1	104.7 ± 1.38	86.5 ± 1.19
Control	100.0 ± 0.71	100.0 ± 1.14
H1	89.8 ± 4.74	108.9 ± 2.68
H2	110.8 ± 1.98	121.3 ± 8.79
H3	129.4 ± 9.92	137.6 ± 8.95
H4	100.4 ± 3.75	107.5 ± 2.12
H5	138.2 ± 6.40	108.8 ± 0.62
rhTGF-β1/H3	112.2 ± 3.31	103.9 ± 0.53

**Table 3:** % Cell proliferation results of formulations (*n* = 3).

	% Cell Proliferation Average ± SD	
Formulation Code	NIH-3T3 Cell Line	HACAT Cell Line
F np	192.6 ± 6.59	81.9 ± 6.74
rhTGF-β1	106.7 ± 9.83	63.5 ± 1.72
Control	100.0 ± 6.44	100.0 ± 1.40
H1	192.6 ± 16.53	75.1 ± 6.97
H2	213.9 ± 26.61	77.2 ± 8.48
H3	240.4 ± 39.10	131.4 ± 34.32
H4	172.8 ± 16.62	90.5 ± 4.82
H5	178.8 ± 18.37	273.4 ± 13.57
rhTGF-β1/H3	199.8 ± 16.71	109.5 ± 6.57

## Data Availability

The original contributions presented in this study are included in the article. Further inquiries can be directed to the corresponding author.

## Abbreviations

PLGA	Poly(Lactic-co-glycolic Acid)
PVA	Polyvinyl Alcohol
DCM	Dichloromethane
FT-IR	Fourier Transform Infrared Spectroscopy
SEM	Scanning Electron Microscopy
rhTGF-β1	Recombinant Human Transforming Growth Factor β-1
BrdU	Bromodeoxyuridine
NPs	Nanoparticle(s)
F	3% PLGA nanoparticle
PVA/Chi	PVA/Chitosan Hydrogels
Control	Ctrl
